# Lifestyle Effects on the Risk of Transmission of COVID-19 in the United States: Evaluation of Market Segmentation Systems

**DOI:** 10.3390/ijerph18094826

**Published:** 2021-04-30

**Authors:** Esra Ozdenerol, Jacob Seboly

**Affiliations:** 1 Spatial Analysis and Geographic Education Laboratory, Department of Earth Sciences, University of Memphis, Memphis, TN 38152, USA; 2 Department of Geosciences, Mississippi State University, Starkville, MS 39762, USA; jds1565@msstate.edu

**Keywords:** geographic information systems, lifestyle segment, Lifemodes, market segmentation, market intelligence, transmission risk, COVID-19 infection, risk mapping

## Abstract

The aim of this study was to associate lifestyle characteristics with COVID-19 infection and mortality rates at the U.S. county level and sequentially map the impact of COVID-19 on different lifestyle segments. We used analysis of variance (ANOVA) statistical testing to determine whether there is any correlation between COVID-19 infection and mortality rates and lifestyles. We used ESRI Tapestry LifeModes data that are collected at the U.S. household level through geodemographic segmentation typically used for marketing purposes to identify consumers’ lifestyles and preferences. According to the ANOVA analysis, a significant association between COVID-19 deaths and LifeModes emerged on 1 April 2020 and was sustained until 30 June 2020. Analysis of means (ANOM) was also performed to determine which LifeModes have incidence rates that are significantly above/below the overall mean incidence rate. We sequentially mapped and graphically illustrated when and where each LifeMode had above/below average risk for COVID-19 infection/death on specific dates. A strong northwest-to-south and northeast-to-south gradient of COVID-19 incidence was identified, facilitating an empirical classification of the United States into several epidemic subregions based on household lifestyle characteristics. Our approach correlating lifestyle characteristics to COVID-19 infection and mortality rate at the U.S. county level provided unique insights into where and when COVID-19 impacted different households. The results suggest that prevention and control policies can be implemented to those specific households exhibiting spatial and temporal pattern of high risk.

## 1. Introduction

As the U.S. healthcare system moves closer to a value-based approach, there is a constantly growing need for market intelligence that provides healthcare providers, health plan providers, major employers and policy-makers with insights into their constituents and positions them to anticipate their needs and behaviors. This study demonstrates the use of market intelligence tools (i.e., lifestyle segments, market segmentation, geodemographic segmentation) to identify a population, determine its lifestyle clusters, and estimate their propensity for various diseases. Throughout this paper, we will use the terms lifestyle segments, lifestyle segmentation, market segmentation, and geodemographic segmentation interchangeably, but they convey the same meaning. Owing to the pandemic, we chose to do our analysis with COVID-19 as a heath outcome. This paper offers a unique spatial and temporal approach proving indispensable for timely and effective ways of analyzing the impact of COVID-19 on American households by their lifestyle characteristics which are summarized as LifeMode groups based on lifestyle and lifestage. We specifically tested two hypotheses: “Is there a difference in average COVID-19 rate among different LifeModes?” and “Which LifeModes have COVID-19 rate that are higher/lower than average?”. We focused on comparing each LifeMode’s mean to the nation’s mean to see spatial and temporal patterns of high risk and the lifestyle effects on the risk of transmission of COVID-19 in the United States.

Lifestyle is an important factor, along with genes, behavior, and environment, that influences humans’ health and their risk for diseases. Lifestyle data are collected at the household level through geodemographic segmentation typically used for marketing purposes to identify consumers’ lifestyles and preferences by private sector marketing [1]. Since this information is used by firms to identify new customers and potential business locations, geodemographic segmentation is a common marketing strategy that involves grouping potential customers into lifestyle segments by state, region, city, or neighborhood.

Reflecting the diversity among American neighborhoods, lifestyle segments reflect demographic shifts over the last decade to established consumer markets, as well as the emergence of new markets due to population growth, geographic, demographic, and socioeconomic change. When composing lifestyle segments, neighborhoods are classified into unique segments nested under LifeMode groups based not only on demographics but also on socioeconomic and behavioral characteristics. For example, geographic data represent where the focal groups are located and where they are buying and using products. Behavioral data focus on when the groups are more likely to buy, under what circumstances the groups are more likely to buy, and how the groups choose to consume or use the product. Demographics represent the races, gender, age groups and marital status of customer/consumers. Psychographic data concentrate on their uniqueness, personal preferences, and lifestyle choices; what they do in their spare time and what products they chose to free up more spare time; how they see themselves and their communities; and identify careers, opinions, and income parameters [2].

Considering the geographic heterogeneity of the U.S. population or the ways in which uniquely characterizing households and their lifestyle, such as retirement communities or diverse urban immigrant enclaves, our approach may provide actionable information for key stakeholders with respect to the focus of interventions and reveal the underlying factors involved in differential health outcomes. Furthermore, such an understanding of the COVID-19 crisis may be instrumental in the implementation of prevention and control policies to those specific households exhibiting spatial and temporal patterns of high risk and may prepare us for future pandemics affecting populations with different lifestyles.

## 2. Materials and Methods 

### 2.1. Data

We combined data from multiple sources and merged them in GIS to create a visual representation through maps. We used the ESRI Tapestry segmentation system [3] to associate lifestyle clusters to COVID-19. The COVID-19 data set is twofold: infection and mortality rates. We explicitly described both ESRI Tapestry segmentation and COVID-19 datasets under separate headings below.

With the advance of GIS technology and cloud computing, progressively better data sets and tools have become available to improve the identification of market segments and the operationalization of a lifestyle segment scheme in the marketplace. Lifestyle segmentation (i.e., market segmentation) describes the division of a market into homogeneous groups, which will respond differently to promotions, communications, advertising, product, pricing, and other marketing mix variables. Examples of the most common commercial or market segmentation systems available today include MOSAIC, ACORN, ESRI Tapestry and many others [3,4,5,6]. These segmentation systems utilize consumer surveys (e.g., Experian’s Consumer View database) and apply traditional customer profiling techniques such as relationships between purchased products and consumers’ beliefs and life patterns [7]. We used the ESRI Tapestry segmentation system [3] that is available on an annual basis as population and household counts by Tapestry segment are updated each year.

#### 2.1.1. ESRI Tapestry Segmentation System

The ESRI Tapestry segmentation system uses Experian’s Consumer View database, the Survey of the American Consumer from GfK MRI [8], and the US Census American Community Survey [9]. The GIS that supports the ESRI Tapestry Segmentation platform illustrates relationships, connections, and visual patterns that are not necessarily obvious in any one data set and enables different demographic data sets to be brought together to create a complete picture of local communities and neighborhoods across the U.S. [3,10].

ESRI tapestry segmentation classifies U.S. neighborhoods into 67 unique market segments, based on socioeconomic and demographic factors, then consolidates these 67 segments into 14 LifeModes with names such as “High Society”, “Senior Styles”, and “Factories and Farms” that have commonalities based on lifestyle and life stage [3]. ESRI Tapestry Segmentation data were downloaded from ESRI [3]. Our dataset contains a variable denoting the dominant tapestry segment within each U.S. county. Table 1 shows the number of counties within each LifeMode. Supplemental material shows a description of the traits of the LifeModes in a table.

#### 2.1.2. COVID-19 Infection and Mortality Rates 

To associate lifestyle clusters to COVID-19, we downloaded two datasets: COVID-19 infection and mortality rates from USAFacts.com [11]. One dataset contained the number of confirmed positive COVID-19 cases in each United States county by date. The other dataset contained the number of COVID-19 deaths in each county by date. These datasets covered the period from 22 January 2020, when the first case in the United States was discovered, to 30 June 2020. Using population data obtained from the U.S. Census Bureau [12], we calculated the rate of infections and deaths per 100,000 residents for each county and date and further mapped the COVID-19 cases by county. This facilitated a more fair and accurate analysis of the impact of COVID-19 on different lifestyles. 

### 2.2. Statistical Methodology

We used analysis of variance (ANOVA) statistical test to determine whether there is any association between COVID-19 infection and mortality rates and lifestyles [13]. We further used analysis of means (ANOM) to determine which lifestyles have higher risk [14]. Since there are many similarities and overlaps between lifestyle segments within the same LifeModes and testing at the segment level would drastically reduce sample sizes—thus curtailing the power of the statistical tests for the statistical analysis—we chose to use the broader tapestry LifeModes, rather than lifestyle segments [3]. Table 2 contains a summary of the statistical tests performed with the corresponding hypothesis tested for the analysis.

Our nationwide analysis included all the counties in the United States. One-way ANOVA was performed with LifeModes as the factor variable and infection rates as the response variable to determine if there is a difference in average infection rate among different LifeModes. The one-way ANOVA compares the means between the LifeModes and determines whether any of those means are statistically significantly different from each other. 

Specifically, it tests the null hypothesis: ‘All means are equal’:

$$H_{o}:\;\mu_{1}=\mu_{2}=\mu_{3}=\cdots =\mu_{k}$$

where *µ* = group mean and *k* = number of groups. If the one-way ANOVA returns a statistically significant result, we accept the alternative hypothesis (H_A_), ‘Not all means are equal’, which means that there are at least two group means that are statistically significantly different from each other.

For infection rates, we ran the ANOVA analysis once for each date during the data time frame. For every date between 1 March and 30 June 2020, the ANOVA analysis revealed a statistically significant association between COVID-19 infections and LifeMode classification. We then performed the same analysis with mortality rates for each day in the period. According to the ANOVA analysis, a significant association between COVID-19 deaths and LifeModes emerged on 1 April 2020 and was sustained through to 30 June 2020.

As some of the assumptions for the one-way ANOVA test were violated, specifically the normality of residuals and homogeneity of variances, we also performed the Welch ANOVA to increase confidence in the results. The Welch ANOVA test confirmed the statistical significance of the association between COVID-19 infections/deaths and LifeModes, with strong *p*-values ranging from 10^−4^ to 10^−40^ depending on the date.

Analysis of means (ANOM) was also performed to determine which LifeModes have incidence rates that are significantly above/below the overall mean incidence rate. For each LifeMode and each date, we calculated a confidence interval for the mean COVID-19 infection rate. For each LifeMode with a confidence interval on a date which was entirely above the overall mean infection rate on that date, we can conclude that this LifeMode had an above average risk of COVID-19 infection on that date. Conversely, for each LifeMode with a confidence interval on a date which was entirely below the overall mean for that date, we can conclude that this LifeMode had a below average risk of COVID-19 infection on that date. The same process was used to determine which LifeModes had above and below average risks of COVID-19 death on which dates. 

## 3. Results 

### 3.1. COVID-19 Spread in the United States

**Figure 1:**
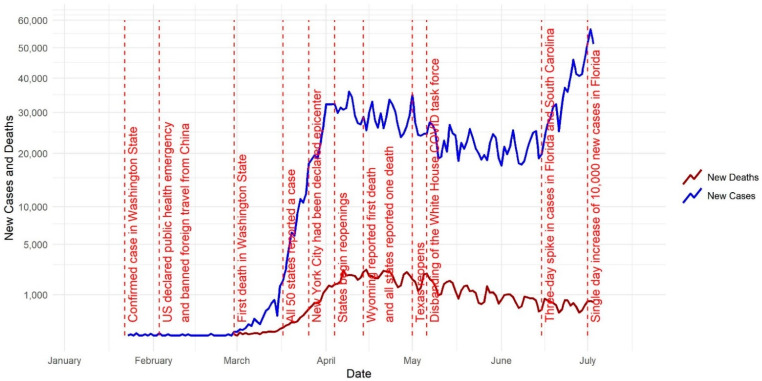
New COVID-19 cases and deaths in conjunction with the timeline of COVID-19 developments.

**Figure 2:**
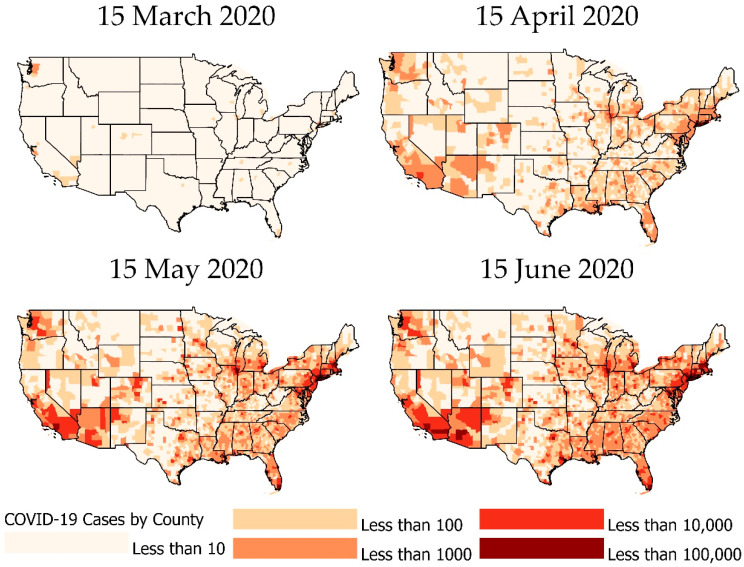
The number of cumulative COVID-19 cases per county from 15 March to 15 June 2020.

In this visual COVID-19 timeline (Figure 1), we delve into some significant milestones that occurred in the United States in 2020 and further mapped COVID-19 cases and deaths (Figure 2). The first cases of a severe respiratory illness that would come to be known as SARS-CoV-2 or COVID-19 were reported in Wuhan, China in December of 2019 [15]. Due to the interconnectedness of the modern world, it took only 21 days for the first reported case of COVID-19 in the United States. The first case of COVID-19 was confirmed in Washington state on 21 January 2020 [16]. The United States declared a public health emergency on 3 February 2020 and banned foreign individuals who had recently traveled to Wuhan from entering the U.S. The first death in the United States was recorded in the state of Washington on 29 February 2020. By 17 March 2020, all 50 states had reported a case [17]. Figure 2 shows the number of cumulative COVID-19 cases per county on 15 March 2020. 

Upon reaching the milestone of having COVID-19 present in every state, it was estimated that more than 6300 cases had been diagnosed in the U.S. and that the global death toll had surpassed 7900 [18]. By 26 March, New York City had been declared the epicenter of the outbreak with more than 20,000 cases, and by 28 March 2020 there were more than 115,000 cases and 1891 deaths in the United States [19]. By 7 April, 395,926 COVID-19 cases had been reported, with two thirds of cases coming from just eight geographic jurisdictions: New York, New Jersey, Michigan, Louisiana, California, Massachusetts, and Pennsylvania [20]. By 14 April, Wyoming reported its first COVID-19-related death, which meant that every state had reported at least one death [17]. Figure 2 shows the number of cumulative COVID-19 cases per county on 15 April 2020. 

Throughout the summer, there was a consistent rise in cases throughout the United States. This increase coincided with the disbanding of the White House COVID-19 task force, and phased reopening of states. In early April, the U.S. President gave permission to state governors to decide how reopening would function. Texas was one of the first states to decide to reopen in early May [17]. Figure 2 shows the number of cumulative COVID-19 cases per county on 15 May 2020. By mid-June, Florida and South Carolina were both recording a three-day long spike in cases. Later, on 2 July, Florida reported a single day increase of 10,000 new cases, and by 8 July, hospitals in both Florida and Arizona began to reach capacity due to their COVID-19 case load [17]. Figure 2 shows the number of cumulative COVID-19 cases per county on 15 June 2020. 

### 3.2. High Risk LifeModes

The results of Welch ANOVA analysis show statistically significant associations between LifeModes and COVID-19 infection rate and COVID-19 mortality rate for all dates in the study period. *p*-values ranged from 10^−4^ to 10^−40^ depending on the date. Table 3 shows the high-risk LifeModes for infection and death.

**Figure 3:**
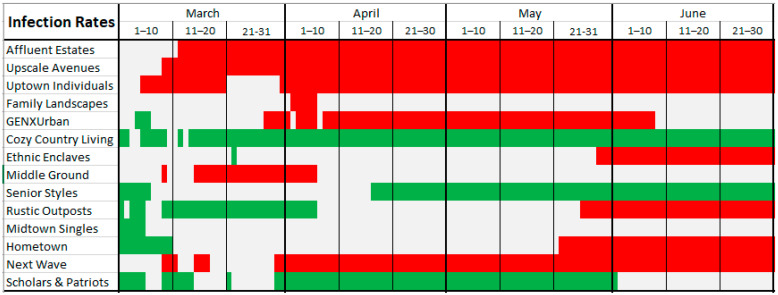
COVID-19 infection risk by LifeMode and date. Red color indicates high risk for that LifeMode and date; green color indicates low risk for that LifeMode and date; white color indicates inconclusive risk.

**Figure 4:**
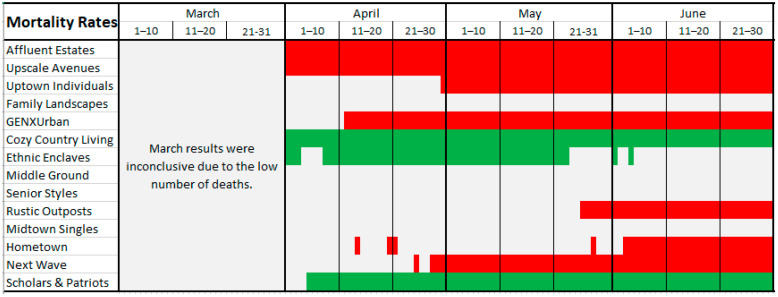
COVID-19 mortality risk by LifeMode and date. Red color indicates high risk for that LifeMode and date; green color indicates low risk for that LifeMode and date; white color indicates inconclusive risk.

Figure 3 and Figure 4 graphically illustrate when each LifeMode had above/below average risk for COVID-19 infection/death on what dates. Figure 3 represents infection rates. LifeModes are displayed on the vertical axis of these charts, while dates are displayed on the horizontal axis. Where each LifeMode intersects with a date, the color of the appropriate grid point represents the risk for that LifeMode on that date. Red represents above average risk, green represents below average risk, and gray represents inconclusive results.

In the cases where a particular LifeMode’s status as high/average/low risk changes over time, we emphasize that the change is not caused by any intrinsic changes in the makeup of the groups, but instead by changes in the relative behavior, mobility, and exposure of the groups. The following paragraphs provide examples and speculation as to how this might be the case with explanations of Figure 3 and Figure 4.

According to Figure 3, *Affluent Estates*, *Upscale Avenues*, and *Uptown Individuals* exhibited a consistently high risk of COVID-19 infection from early March through to June. These are more affluent people who travel much more than the average American and are active in fitness pursuits such as bicycling, jogging, yoga, and hiking. *Next Wave* also exhibited high risk from late March through to June. These are mostly people of international origin and immigrants who frequently travel overseas. Thus, travel may explain why these lifestyles were more likely to be exposed to COVID-19 in the early stages of the pandemic. *GenXUrban* became a high-risk LifeMode in late March but went back to inconclusive in early June. The households in this LifeMode are generally 38–47 years old, married, living in single family housing, are predominantly white, and make USD 47,000–68,000 annually. They own older single-family homes in urban areas, live and work in the same county, enjoy going to museums and rock concerts, dining out, and walking for exercise. 

LifeModes representing poorer households, including *Ethnic Enclaves*, *Rustic Outposts*, and *Hometown*, became high risk in late May and stayed in that category through the rest of our study period. While members of these LifeMode segments do not travel as often, they are more likely to work at essential (blue-collar) jobs, less likely to have the opportunity to work from home, and probably have less access to quality healthcare. Members of these LifeModes, such as Latino communities of *Ethnic Enclaves,* may have also been more skeptical of government and public health recommendations regarding COVID-19 safety and afraid to seek out public services, including medical care [21]. All these factors could have combined to ensure that these lifestyles became high-risk once the pandemic was more established in the United States. 

LifeModes which have remained consistently at low risk throughout the pandemic include *Cozy Country Living, Senior Styles*, and *Scholars and Patriots*. *Cozy Country Living* represents the most rural LifeMode. It is found to be low risk for both infection and death. These are the last communities that the pandemic reached, as these are the most disconnected from urban society and international travel. *Senior Styles* represents mostly retirement communities, nursing homes, and neighborhoods where the population is predominantly made up of those over 65. Given the elevated risk of COVID-19 to the elderly, these communities generally took extra precautions to prevent the spread of COVID-19, explaining their low risk of infection. *Senior Styles* (9) is low risk for infection only, but not for death. *Scholars and Patriots* communities consist mainly of university towns and military bases. Both universities and military bases took significant steps to prevent outbreaks of COVID-19, with university towns even sending many of their students home. This would have limited opportunities for COVID-19 to affect these LifeModes. 

Figure 4 is drawn according to the same scheme but represents mortality rates instead. According to Figure 4, the analysis of mortality rates yields many of the same results as the infection rate analysis. However, *Ethnic Enclaves* was high risk for infection but low risk for death. This might be observed because *Ethnic Enclaves* mostly consists of younger families, and COVID-19 is much less likely to cause death in young and middle-aged people. Meanwhile, *Senior Styles* is low risk for infection but not for death. This occurs because the mortality rate for the elderly is much higher, so even if these populations see fewer than average cases, they can still reach an average rate of deaths. 

### 3.3. Spatial Variation of High-Risk Life Modes

**Figure 5:**
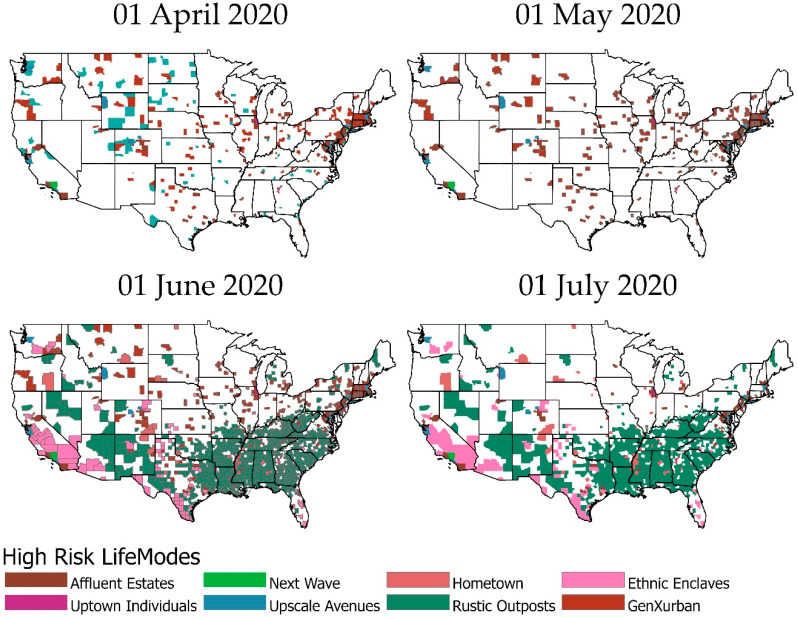
High-risk LifeModes from 1 April to 1 July 2020.

We mapped the high-risk life modes associated with COVID-19 incidence and mortality. These maps demonstrate that the lifestyle characteristics of these LifeModes account for some of the spatial variation and significantly contribute to viral transmission and incidence/mortality rates. According to Figure 5, *Affluent Estates, Upscale Avenues*, and *Uptown Individuals* exhibited consistently high risk of COVID-19 infection during the pandemic’s early days in March and throughout April in the west coast cities. *Upscale Avenues* were the first and the most impacted by COVID-19 in Seattle, Washington and Los Angeles, CA. *Upscale Avenues* was also the first group impacted by COVID-19 in the states of Wyoming and Colorado, which are both vacation destinations and retirement communities of these prosperous LifeModes.

*GenXUrban* was only impacted in Washington and Oregon states early in the pandemic in inland areas of these states. It is possible that they may have been exposed to a returned traveler who was infected. *GenXUrban* became the most dominant LifeMode in less dense areas of inland states in March and April. Our results indicate that *GenXUrban* (5) is a high-risk LifeMode for death only. Therefore, this merits attention as to what makes this LifeMode high risk for death. They could face health challenges such as lack of health care and social services. *Affluent Estates* were the most impacted by COVID-19 in San Francisco, Los Angeles, and San Diego in California, and major east coast cities such as New York, Boston, and Washington D.C., where the virus arrived early and spread quickly. These dynamics of the outbreak swept through American cities in early March. We see a hierarchical spread of the pandemic from the coastal gateway cities to major cities of inland areas by plane and by car travel. For example, Lake Tahoe is a weekend getaway for vacationers, and this explains the hotspot of the members of *Affluent Estates* in this area and the transmission to the *GenXUrban* members of the local community.

The *New Wave* LifeMode, consisting of frequent overseas travelers, was also a dominant life mode in Los Angeles in Southern California that has been the most impacted and exposed to COVID-19 in the early stages of the pandemic. The *New Wave* LifeMode is the most racially and ethnically diverse among LifeMode groups with a Hispanic majority. A large share is foreign born and speak only their native language. Most are renters in older multi-unit structures and live-in crowded homes. *Uptown Individuals* only exhibited in Chicago and Atlanta, as they are the gateway cities of international travel and represent young successful singles that are also frequent travelers and reside in highly dense cities. This LifeMode represents a younger population in comparison to the prosperous married couple members of *Affluent Estates* and *Upscale Avenues*. This pattern continued through to June.

As states moved to ease lock down restrictions and reopened in May, new LifeModes emerged as being impacted by COVID-19 and the growth subsumed rural areas of California, Texas, and southern states. This time, LifeModes represented poorer households, including *Ethnic Enclaves, Rustic Outposts*, and *Hometown*. The members of these LifeModes are more likely to work at essential (blue-collar) jobs, less likely to have the opportunity to work from home and have less access to quality healthcare. Young, Hispanic families, multilingual and multigenerational households of *Ethnic Enclaves* in Texas and Southern Florida became targets of COVID-19. The *Hometown* LifeMode was impacted by COVID-19 in Native American communities in New Mexico, Colorado, and Oregon. COVID-19 hit the poor communities of the Mississippi River delta and impacted members of the *Hometown* LifeMode. *Rustic Outposts* emerged as a dominant Life Mode in the entire southern United States impacted by COVID-19, and rural areas became high risk.

**Figure 6:**
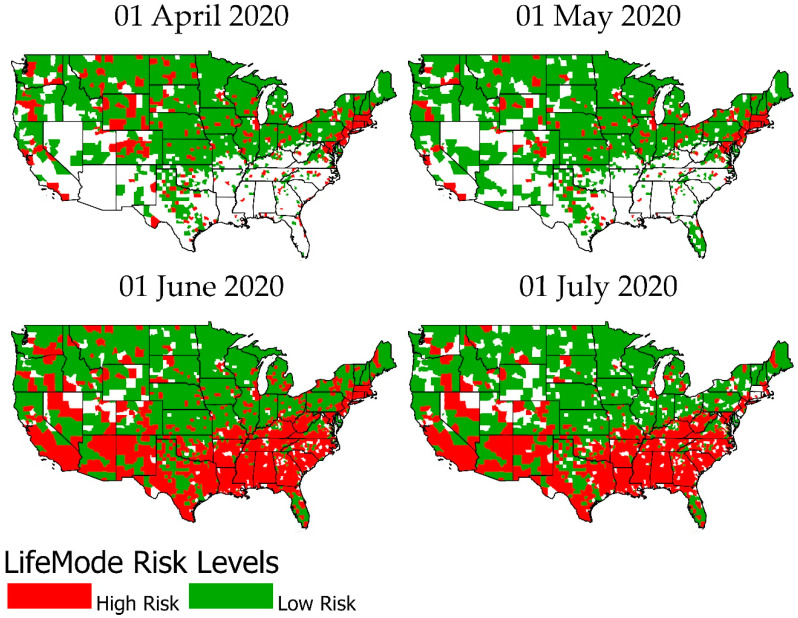
High- and low-risk LifeModes from 1 April to 1 July 2020.

Figure 6 shows the shift from no outbreak to low risk to high risk LifeModes quickly spreading and covering the coastal and southern counties of the nation from the beginning of the outbreak in March to July. Table 4 shows that the mean case rate by life mode is highest among *Next Wave* (1965), *Uptown Individuals* (1865), *Upscale Avenues* (1479), *Midtown Singles* (1429), *Hometown* (1387), and lowest among *Scholars* *and Patriots* (555) and *Cozy Country Living* (477). Contrary to the high mortality rates of *Senior Styles,* this LifeMode was low risk for infection only in comparison to *Ethnic Enclaves,* which was high risk for infection only. *GenXUrban* was high risk for death only, while *Scholars and Patriots* was low risk for death only.

### 3.4. Demographic Profile of High-Risk LifeModes

We analyzed the resultant high-risk LifeModes by their demographic profile, specifically by their diversity index, population density, population by age groups, college education, median age and household size (see Table 5).

The diversity index summarizes racial and ethnic diversity. The index shows the likelihood that two persons, chosen at random for the same area, belong to different racial or ethnic groups. The index ranges from 0 (no diversity) to 100 (complete diversity). For example, the diversity core for the whole of the U.S. is 64.0, which means there is a 64.0 percent probability that two people randomly chosen from the US population would belong to a different race or ethnic group. Looking at the demographic profile of high-risk LifeModes for COVID-infection, the diversity index is found to be high among high risk LifeModes. For example, the *New Wave* LifeMode has a diversity index of 89.5, followed by *Ethnic Enclaves* (82.4) and *Midtown Singles* (78.4). These LifeModes are urban neighborhoods in which immigrant groups or ethnic minorities are residentially concentrated. In the COVID-19 literature, people residing in immigrant neighborhoods were less likely to be tested; but the likelihood that a test was positive was larger in those neighborhoods, as well as in neighborhoods with larger households or predominantly Black populations. COVID-19 diagnoses were associated with counties with greater monolingual Spanish speakers [21,22,23].

We see more cases of COVID-19 and high mortality rates in high-risk LifeModes with high population densities. The *Uptown Individuals* (7614.5), *Next Wave* (4252.3), *Midtown Singles* (2398.5) and *Upscale Avenues* (1105.5) LifeModes have high COVID-19 mortality rates and they reside in highly dense urban areas and live in higher-density housing. The *GenXurban* LifeMode, which is found to be high risk for death only, has the highest percent population of 65 and above (20.4%), followed by 45–64 at 27.9%. This LifeMode has a growing population of retirees. In contrary, *Ethnic Enclaves*, which is high risk for infection only, has the high percentage of the population below 18 years of age at 29.1%, followed by 18–44 at 40%. *Ethnic Enclaves* also stands out by average household size being 3.35, followed by *Next Wave* (3.31). *Scholars and Patriots* is low risk for death only and has the lowest population of 65 and above at 4.8%. The population of this LifeMode has the highest proportion of individuals between 18 and 44 at 76.9%, including college campuses and military populations. College enrollment is highest among members of *Scholars and Patriots* (49.5), followed by *Midtown Singles* (7.9) and *Uptown Individuals* (6.8). *Senior Styles* are low risk for infection only, with a high percentage of 65 and above population (37.9%). *Senior Style* households are commonly married empty nesters or singles living alone; homes are single-family (including seasonal getaways), retirement communities, or high-rise apartments.

### 3.5. Economic Profile of the High-Risk LifeModes

When we review the economic profile of the high-risk LifeModes in Table 6, members of *Affluent Estates* (31.7), *Upscale Avenues* (29.5), and *Uptown Individuals* (36.9) that exhibited consistently high risk of COVID-19 infection during the pandemic’s early days were professionals by occupation, in comparison to *Next Wave* (30.4), *Hometown* (25.1), *Midtown Singles* (26.5), who engage primarily in service occupations. Table 7 shows that the unemployment rate is highest among *Hometown* (9.5), *Midtown Singles* (8.0) and *Next Wave* (7.8). The service industry is the industry with the highest percentage of members of high risk LifeModes employed, followed by the finance, real estate and retail trade industries. *Next Wave* (9.8), *Rustic Outposts* (8.9), *Ethnic Enclaves* (8.3) *and Cozy Country Living* (8.0) are the high-risk life modes that have the highest percentage of employment in the construction industry.

## 4. Discussions

During a time of increased attention on social determinants of health (SDOH), our finding that human social behaviors such as lifestyle preferences affect the risk of contracting COVID-19 proved very useful in comparison to the index-based approaches that quantify SDOH. With this increased understanding of SDOH and with market intelligence tools, we can target vulnerable populations that could be impacted by COVID-19 for prevention and control strategies. Lifestyle segmentation classifies neighborhoods into unique segments (i.e., 67 segments) and LifeMode groups (i.e., 14 summary groups) based not only on demographics but also socioeconomic and behavioral characteristics. With a greater depth of understanding of these at-risk households based on lifestyle, we can explore the localized households and predict expansion of the geographic spread of the pandemic. These households can be targeted for better social services for COVID-19 at clinical settings. Public health messages and clinical information could be issued to the public and medical practitioners for these at-risk households to provide better assistance in clinical diagnoses and to implement preventive measures such as social distancing and reducing unnecessary travel. This paper makes a unique contribution to the public health literature by associating lifestyle characteristics to COVID-19 infection and mortality rates at the U.S. county level and sequentially mapping the impact of COVID-19 on different lifestyles.

Even though SDOH are broadly defined by the World Health Organization as the conditions in which people are born, grow, live, work and age [24], the phenomena are often represented solely by socioeconomic indicators, such as income and education. SDOH indicators, such as income, are associated with greater life expectancy in the United States; however, these associations may change based on the underlying area characteristics and health behaviors [25]. Index-based approaches are used as proxy methods to quantify SDOH [26,27,28,29]. The area deprivation index (ADI) by Singh et al. [26,27,28], which was extended by Kind et al. [29], focused on socioeconomic disadvantage and the differing dimensions of poverty. The SDOH index by The Carolinas Health Care system includes additional dimensions such as food accessibility, though these have not yet been validated against actualized health outcomes and cannot reveal the underlying factors involved in differential health out-comes [30]. Kolak et al. [31] modeled SDOH as multivariate indices rather than as a singular deprivation index. Using variables of advantage, isolation, opportunity, mixed immigrant cohesion and accessibility, they imported their findings into seven distinct multidimensional neighborhood typologies. Even though they attempted to address spatial heterogeneity, their data only allowed for cross-sectional analyses, which introduced the risk of missing changes in socioeconomic patterns at the census tract level and associated health outcomes over time.

These index-based approaches that quantify SDOH overlook the geographic heterogeneity of the U.S. population or the ways in which uniquely characterized households and their lifestyle, such as retirement communities or diverse urban immigrant enclaves behave and contribute to spread of the virus. We addressed this challenge with market intelligence tools (i.e., lifestyle segments, market segmentation) by identifying a population, determining its lifestyle clusters, and estimating their propensity for COVID-19 infection and death.

SDOH indicators have different associations with differing health outcomes. SDOH interacts with health outcomes differently in different places [31]. Through a scoping analysis of the literature on COVID-19 and SDOH, we identified studies that have thus far found SDOH associated with COVID-19 transmission and mortality [32,33,34,35,36,37]. COVID-19 is disproportionally impacting certain populations according to race-ethnicity and socioeconomic status [10,11,12,13,14,15,16,17,18]. Large disparities across race-ethnicity and socioeconomic status exist in the prevalence of conditions which are associated with the risk of severe complications from COVID-19 [32,33]. Counties with higher proportions of Black people have a higher prevalence of comorbidities and had more COVID-19 diagnoses and deaths, after adjusting for county-level characteristics such as age, poverty, comorbidities, and epidemic duration [32]. COVID-19 deaths were higher in disproportionally Black rural and small metro counties [32]. Li et al. also found that U.S. counties with a higher proportion of Black residents are associated with increased COVID-19 cases and deaths; however, the various suggested mechanisms, such as socioeconomic and healthcare predispositions, did not appear to drive the effect of race in their model [33]. Counties with higher average daily temperatures are also associated with decreased COVID-19 cases but not deaths.

Several theories are posited to explain these findings, including the prevalence of vitamin D deficiency [33]. While vulnerability is highest among older adults regardless of their race-ethnicity or socioeconomic status, the findings suggest particular attention should also be given to the risk of adverse outcomes in midlife for non-Hispanic Blacks, adults with a high school degree or less, and low-income Americans [34,35,36]. COVID-19 diagnoses rates were greater in Latino counties nationally. In multivariable analysis, COVID-19 cases were greater in Northeastern and Midwestern Latino counties. COVID-19 deaths were greater in Midwestern Latino counties. COVID-19 diagnoses were associated with counties with greater monolingual Spanish speakers, employment rates, heart disease deaths, less social distancing, and days since the first reported case. COVID-19 deaths were associated with household occupancy density, air pollution, employment, days since the first reported case, and age (fewer <35 y.o.) [21]. Studies modelling and mapping intercounty transmission risk of COVID-19 in New York City merged information on the number of tests and the number of infections at the zip code level with demographic and socioeconomic information from the decennial census and the American Community Surveys. The rate of infection in the population depends on both the frequency of tests and on the fraction of positive tests among those tested. People residing in poor or immigrant neighborhoods were less likely to be tested, but the likelihood that a test was positive was larger in those neighborhoods, as well as in neighborhoods with larger households or predominantly Black populations [22,23].

In addition to race-ethnicity and socioeconomic status, some studies focused on other SDOH indicators. A study in Germany found the strongest predictors of COVID-19 incidence at the county scale were related to community interconnectedness, geographical location, transportation infrastructure, and labor market structure [37]. Location, densities of the built environment, and socioeconomic variables are important predictors of COVID-19 incidence rates; however, these SDOH associations are complex and may change based on underlying area characteristics and lifestyle behaviors.

This paper contributes to the SDOH and COVID-19 literature by offering a unique spatial and temporal approach that proves indispensable for timely and effective way of analyzing impact of COVID-19 on American households by their lifestyle characteristics. We demonstrated that COVID-19 was first introduced to West Coast Metropolitan cities by frequently traveling LifeModes such as *Affluent Estates* and *Uptown Individuals* and then was introduced through community spread to *GenXUrban* and successively poorer American households, including *Ethnic Enclaves*, *Rustic Outposts*, and *Hometown* segments. Our findings reveal that the affluent and mobile lifestyles exhibited highest risk of infection during the early stages of the outbreak, then the risk shifted to the poor, isolated, and vulnerable lifestyles during the mature stages.

The most important limitation is that this is a population study. The dominant LifeMode in a county does not represent every single household in that county; each county contains a unique mixture of all fourteen LifeModes. Additionally, while Tapestry descriptions provide accurate representations of today’s consumers, they are generalizations about consumers with no guarantee that a given household will fit perfectly into a specific segment or LifeMode. Rather, Tapestry segmentation is best used as a set of common characteristics among the typical consumer household. LifeMode groups represent markets that share a common experience—born in the same generation or immigration from another country—or a significant demographic trait, such as affluence. All the consumers in the proposed county will not fall into a specific LifeMode summary group but a representative one, composed of a population—a complete set of people with a specialized set of consumer characteristics. Our ultimate decision on using the dominant LifeMode so that the results of our study can be generalized to a larger population such as county depends on this understanding.

## 5. Conclusions

We can only build a culture of health by engaging health and related non-health sectors. The geographic information science coupled with health information technology can help to develop links between the two sectors that match lifestyle segments from private sector marketing data to those of geographically identified patients in healthcare delivery systems. By correlating COVID-19 data with specific lifestyle segments, it becomes possible to identify patterns of transmission and predict the demand and utilization of health services. 

We conclude that there needs to be more research done to translate scientific data into real world solutions. Given that virtually every household in the U.S. has been assigned a lifestyle segment, linking segments to geographically identified patients (e.g., incidence, morbidity) in health care delivery systems could support the ability to estimate morbidity levels for COVID-19 and subsequently predict the demand for health services. Such an understanding of the COVID-19 crisis could be instrumental in better preparing us for a future pandemic or future wave, as it could be used to predict how future epidemics would be introduced to American households, particularly in the early stages of the outbreak with the affluent and mobile lifestyles, or during late stages with the poor, isolated and vulnerable lifestyles.

Our approach may provide actionable information for key stakeholders with respect to the focus of interventions and reveal the underlying factors involved in differential health outcomes. Sequentially mapping and geographically illustrating when and where each LifeMode had above/below average risk for COVID-19 infection/death provided clues regarding at-risk households and the timing of their infection and possible intervention strategies for future scenarios.

As the U.S. healthcare system moves closer to a value-based approach, there will be a constantly growing need for market intelligence that provides healthcare providers, health plans, major employers and policy makers with insights into their constituents and positions them to anticipate their needs and behaviors. This could be accomplished by identifying a population, determining its lifestyle clusters, and estimating the propensity for various diseases. Targeting key lifestyle modes/segments and their locations enables the health care system to focus on communities that might have been missed when straight demographic criteria were used.

## Figures and Tables

**Table 1:** Dominant LifeMode within each U.S. county.

LifeMode Name	Code	Counties
Affluent Estates	1	71
Upscale Avenues	2	41
Uptown Individuals	3	13
Family Landscape	4	159
GenXUrban	5	163
Cozy Country Living	6	1261
Ethnic Enclaves	7	106
Middle Ground	8	80
Senior Styles	9	69
Rustic Outposts	10	965
Midtown Singles	11	21
Hometown	12	141
Next Wave	13	5
Scholars and Patriots	14	45
Total	14	3140

**Table 2:** Summary of statistical tests performed and research hypotheses.

Test	Research Hypotheses
ANOVA/Welch ANOVA	Is there a difference in average COVID-19 rate among different LifeModes?
ANOM	Which LifeModes have COVID-19 rates that are higher/lower than average?

**Table 3:** High-risk LifeModes for COVID-19 infection and death.

Affluent Estates (1)
Upscale Avenues (2)
Uptown Individuals (3)
GenXUrban (5) *
Ethnic Enclaves (7) **
Rustic Outposts (10)
Hometown (12)
Next Wave (13)

* GenXUrban (5) is high risk for death only. ** Ethnic Enclaves (7) is high risk for infection only.

**Table 4:** Mean case rate by LifeMode.

LifeMode	Code	Counties	Mean Infection Rate * (Cases per 100,000)	Mean Mortality Rate * (Deaths per 100,000)
Affluent Estates	1	87	(766, 1134)	(39.4, 62.7)
Upscale Avenues	2	33	(1029, 1966)	(48.5, 102.6)
Uptown Individuals	3	9	(1178, 2084)	(55.2, 131.3)
Family Landscapes	4	111	(584, 742)	(14.4, 22.4)
GenXUrban	5	195	(585, 750)	(26.8, 38.6)
Cozy Country Living	6	1383	(359, 438)	(10.1, 12.5)
Ethnic Enclaves	7	89	(1004, 1729)	(14.0, 23.5)
Middle Ground	8	82	(510, 784)	(11.6, 37.4)
Senior Styles	9	66	(369, 592)	(10.6, 25.0)
Rustic Outposts	10	971	(732, 870)	(23.6, 29.6)
Midtown Singles	11	11	(594, 1645)	(12.2, 107.6)
Hometown	12	65	(812, 1423)	(28.2, 59.3)
Next Wave	13	5	(2308, 4198)	(146.5, 364.1)
Scholars and Patriots	14	35	(367, 974)	(4.6, 13.6)

* 95% confidence interval bounds generated via 1000-member bootstrap ensembles.

**Table 5:** Demographic profile of LifeModes in comparison to overall U.S averages.

	COVID-19 Cases per 100,000 *	COVID-19 Deaths per 100,000 *	Diversity Index	Median Age	Ave. HH Size	Pop Density (Persons per mi^2^)	Pop <18	Pop 18–44	Pop 45–64	Pop 65+	College Enrollment
United States	848	41.00	64	38.2	2.59	92.7	22.3	36.1	25.9	15.6	6.3
Affluent Estates	1114	59.87	43.8	43	2.89	503.2	24.7	28.0	31.2	16.1	4.9
Upscale Avenues	1479	83.56	66.5	40.6	2.70	1105.5	21.1	34.8	28.3	15.9	6.1
Uptown Individuals	1865	109.8	65	34.7	1.86	7614.5	11.7	55.8	21.4	11.2	6.8
Family Landscapes	716	18.12	54	36.8	2.86	294.4	25.4	36.0	26.9	11.7	5.6
GenXUrban	893	52.04	41.2	43.5	2.43	409.8	19.7	32.0	27.9	20.4	5.5
Cozy Country Living	477	17.74	26.6	45	2.53	21.9	20.5	29.5	30.6	19.4	3.9
Ethnic Enclaves	1087	19.05	82.4	31.8	3.35	134.2	29.1	40.0	21.6	9.3	6.1
Middle Ground	1209	88.44	68.9	36	2.41	396.8	21.7	40.2	24.2	13.8	6.7
Senior Styles	842	31.26	47.6	57.4	1.94	89.3	12.3	23.9	25.9	37.9	4.7
Rustic Outposts	818	25.33	49.1	40.7	2.60	29.2	22.0	33.3	28.0	16.7	3.8
Midtown Singles	1429	94.15	78.4	30.9	2.38	2398.5	25.0	45.2	20.4	9.4	7.9
Hometown	1387	89.11	65.5	38	2.48	200.5	23.3	34.8	25.4	16.5	5.4
Next Wave	1965	116.1	89.5	29.8	3.31	4252.3	29.3	43.0	19.8	7.9	5.9
Scholars and Patriots	555	9.04	58.2	22.8	2.28	464.2	10.4	76.9	7.9	4.8	49.5

* COVID-19 data obtained 30 June 2020. Source: ESRI Tapestry [3].

**Table 6:** Employment by occupation (%).

	COVID-19 Cases per 100,000	COVID-19 Deaths per 100,000	Mgmt./Business Financial	Professional	Sales	Admin Support	Services	Farming Forestry Fishing	Constr. Extraction	Installat. Maint. Repair	Production	Transp. Material Moving
United States	848	41.00	14.6	22.2	10.5	13.2	18.5	0.8	4.9	3.2	5.8	6.2
Affluent Estates	1114	59.87	25.1	31.7	12.2	10.6	10.5	0.2	2.6	1.9	2.4	2.9
Upscale Avenues	1479	83.56	19.6	29.5	10.6	12.8	14.5	0.2	3.5	2.4	3.1	3.9
Uptown Individuals	1865	109.8	24.5	36.9	10.0	9.4	12.8	0.1	1.6	1.0	1.6	2.2
Family Landscapes	716	18.12	15.9	23.0	11.0	14.3	15.8	0.4	4.7	3.8	5.4	5.8
GenXUrban	893	52.04	14.5	24.1	10.6	14.4	17.0	0.4	4.4	3.3	5.5	5.6
Cozy Country Living	477	17.74	13.8	19.1	9.5	13.1	16.7	1.6	6.3	4.4	8.2	7.4
Ethnic Enclaves	1087	19.05	10.5	15.7	10.3	14.2	21.2	2.4	6.7	3.9	6.9	8.2
Middle Ground	1209	88.44	12.9	21.6	10.6	13.9	21.2	0.4	4.6	2.9	5.6	6.3
Senior Styles	842	31.26	16.0	23.9	11.8	13.3	19.1	0.6	3.8	2.5	3.9	5.0
Rustic Outposts	818	25.33	9.6	15.3	9.3	13.0	19.0	1.9	8.0	5.2	9.9	8.9
Midtown Singles	1429	94.15	9.7	17.9	10.7	14.7	26.5	0.4	4.6	2.7	5.5	7.2
Hometown	1387	89.11	8.1	15.2	9.8	14.6	25.1	0.6	4.9	3.4	9.1	9.1
Next Wave	1965	116.1	6.3	10.5	9.4	11.9	30.4	1.3	8.7	3.0	8.8	9.7
Scholars and Patriots	555	9.04	9.1	28.0	11.5	14.0	26.1	0.6	2.3	1.8	2.9	3.9

**Table 7:** Employment by Industry (%).

	COVID-19 Cases per 100,000	COVID-19 Deaths per 100,000	Unemp. Rate	Agri Mining	Constr	Manuf.	Wholesale Trade	Retail Trade	Trans. Utilities	Information	Finance Real Estate	Services	Public Admin
United States	848	41.00	5.5	1.9	6.4	10.1	2.6	11.0	5.1	1.8	6.7	50.0	4.5
Affluent Estates	1114	59.87	3.3	0.9	4.8	9.3	3.2	9.0	3.8	2.3	10.1	52.0	4.6
Upscale Avenues	1479	83.56	4.0	0.5	5.3	7.8	2.8	9.6	4.6	2.7	8.3	53.3	5.1
Uptown Individuals	1865	109.8	3.4	0.4	2.7	5.1	2.0	7.4	2.7	4.4	10.4	61.0	4.0
Family Landscapes	716	18.12	4.4	1.3	6.6	10.5	3.0	11.5	5.7	1.8	7.1	47.2	5.4
GenXUrban	893	52.04	4.5	1.1	5.9	10.3	2.6	11.4	4.9	1.6	6.9	50.4	4.9
Cozy Country Living	477	17.74	4.4	4.6	8.0	14.1	2.6	10.9	5.7	1.2	5.2	43.2	4.6
Ethnic Enclaves	1087	19.05	6.6	3.4	8.3	9.4	3.0	11.7	6.3	1.5	5.6	46.4	4.4
Middle Ground	1209	88.44	5.7	0.9	5.8	8.7	2.5	11.8	4.9	1.8	6.6	52.8	4.2
Senior Styles	842	31.26	5.7	1.2	5.4	7.2	2.4	11.3	4.3	1.7	6.7	53.9	4.4
Rustic Outposts	818	25.33	6.7	4.9	8.9	14.3	2.4	11.7	6.3	1.0	8.1	41.7	4.9
Midtown Singles	1429	94.15	8.0	0.7	5.5	7.3	2.1	12.5	5.4	1.7	4.0	54.8	3.9
Hometown	1387	89.11	9.5	1.2	5.5	12.4	2.1	12.2	6.0	1.3	6.1	50.0	4.6
Next Wave	1965	116.1	7.8	1.5	9.8	10.1	2.8	11.2	5.7	1.3	4.7	51.1	2.2
Scholars and Patriots	555	9.04	7.0	0.9	2.7	4.4	1.2	12.3	2.0	1.6	4.3	67.1	3.9

## Data Availability

Not applicable.

## Supplementary Materials

The following are available online at https://www.mdpi.com/article/10.3390/ijerph18094826/s1.
