# Supplementary material for: Lifestyle Effects on the Risk of Transmission of COVID-19 in the United States: Evaluation of Market Segmentation Systems
Source: Int J Environ Res Public Health. 2021 Apr 30;18(9):4826. doi: 10.3390/ijerph18094826 (PMC8125751; doi:10.3390/ijerph18094826)
Supplement: Supplementary file 1 [file ijerph-18-04826-s001.zip › ijerph-1200754-supplementary.pdf]

**Supplementary Materials-Description of Life Modes**

| Life Mode |
|---|
| LifeMode1 Affluent Estates<br><br>• Established wealth—educated, well-traveled married couples<br>• Accustomed to "more": less than 10% of all households, with 20% of household income<br>• Homeowners (almost 90%), with mortgages (65.2%)<br>• Married couple families with children ranging from grade school to college<br>• Expect quality; invest in time-saving services<br>• Participate actively in their communities<br>• Active in sports and enthusiastic travelers |
| LifeMode 2 Upscale Avenues<br><br>• Prosperous married couples living in older suburban enclaves<br>• Ambitious and hard-working<br>• Homeowners (70%) prefer denser, more urban settings with older homes and a large share of townhomes<br>• A more diverse population, primarily married couples, many with older children<br>• Financially responsible, but still indulge in casino gambling and lotto tickets<br>• Serious shoppers, from Nordstrom's to Marshalls or DSW, that appreciate quality, and bargains<br>• Active in fitness pursuits like bicycling, jogging, yoga, and hiking<br>• Subscribe to premium movie channels like HBO and Starz |
| LifeMode 3 Uptown Individuals<br><br>• Young, successful singles in the city<br>• Intelligent (best educated market), hard-working (highest rate of labor force participation) and averse to traditional commitments of marriage and home ownership<br>• Urban denizens, partial to city life, high-rise apartments and uptown neighborhoods<br>• Prefer credit cards over debit cards, while paying down student loans<br>• Green and generous to environmental, cultural and political organizations<br>• Internet dependent, from social connections to shopping for fashion, tracking investments, making travel arrangements, and watching television and movies<br>• Adventurous and open to new experiences and places |
| LifeMode 4 Family Landscapes<br><br>• Successful young families in their first homes<br>• Non-diverse, prosperous married-couple families, residing in suburban or semirural areas with a low vacancy rate (second lowest)<br>• Homeowners (79%) with mortgages (second highest %), living in newer single-family homes, with median home value slightly higher than the U.S.<br>• Two workers in the family, contributing to the second highest labor force participation rate, as well as low unemployment<br>• Do-it-yourselfers, who work on home improvement projects, as well as their lawns and gardens<br>• Sports enthusiasts, typically owning newer sedans or SUVs, dogs, and savings accounts/plans, comfortable with the latest technology<br>• Eat out frequently at fast food or family restaurants to accommodate their busy lifestyle |

| |
|---|
| • Especially enjoy bowling, swimming, playing golf, playing video games, watching movies rented via Redbox, and taking trips to a zoo or theme park |
| LifeMode 5 GenXurban<br><br>• Gen X in middle age; families with fewer kids and a mortgage<br>• Second largest Tapestry group, comprised of Gen X married couples, and a growing population of retirees<br>• About a fifth of residents are 65 or older; about a fourth of households have retirement income<br>• Own older single-family homes in urban areas, with 1 or 2 vehicles<br>• Live and work in the same county, creating shorter commute times<br>• Invest wisely, well-insured, comfortable banking online or in person<br>• News junkies (read a daily newspaper, watch news on TV, and go online for news)<br>• Enjoy reading, renting movies, playing board games and cards, doing crossword puzzles, going to museums and rock concerts, dining out, and walking for exercise |
| LifeMode 6 Cozy Country Living<br><br>• Empty nesters in bucolic settings<br>• Largest Tapestry group, almost half of households located in the Midwest<br>• Homeowners with pets, residing in single-family dwellings in rural areas; almost 30% have 3 or more vehicles and, therefore, auto loans<br>• Politically conservative and believe in the importance of buying American<br>• Own domestic trucks, motorcycles, and ATVs/UTVs<br>• Prefer to eat at home, shop at discount retail stores (especially Walmart), bank in person, and spend little time online<br>• Own every tool and piece of equipment imaginable to maintain their homes, vehicles, vegetable gardens, and lawns<br>• Listen to country music, watch auto racing on TV, and play the lottery; enjoy outdoor activities, such as fishing, hunting, camping, boating, and even bird watching |
| LifeMode 7 Ethnic Enclaves<br><br>• Established diversity—young, Hispanic homeowners with families<br>• Multilingual and multigenerational households feature children that represent second-, third- or fourth-generation Hispanic families<br>• Neighborhoods feature single-family, owner-occupied homes built at city's edge, primarily built after 1980<br>• Hard-working and optimistic, most residents aged 25 years or older have a high school diploma or some college education<br>• Shopping and leisure also focus on their children—baby and children's products from shoes to toys and games and trips to theme parks, water parks or the zoo<br>• Residents favor Hispanic programs on radio or television; children enjoy playing video games on personal computers, handheld or console devices<br>• Many households have dogs for domestic pets |
| LifeMode 8 Middle Ground<br><br>• Lifestyles of thirtysomethings<br>• Millennials in the middle: single/married, renters/homeowners, middle class/working class<br>• Urban market mix of single-family, townhome, and multi-unit dwellings |

- Majority of residents attended college or attained a college degree
- Householders have ditched their landlines for cell phones, which they use to listen to music (generally contemporary hits), read the news, and get the latest sports updates of their favorite teams
- Online all the time: use the Internet for entertainment (downloading music, watching YouTube, finding dates), social media (Facebook, Twitter, LinkedIn), search for employment
- Leisure includes night life (clubbing, movies), going to the beach, some travel and hiking

LifeMode 9 Senior Styles

- Senior lifestyles reveal the effects of saving for retirement
- Households are commonly married empty nesters or singles living alone; homes are single-family (including seasonal getaways), retirement communities, or high-rise apartments
- More affluent seniors travel and relocate to warmer climates; less affluent, settled seniors are still working toward retirement
- Cell phones are popular, but so are landlines
- Many still prefer print to digital media: Avid readers of newspapers, to stay current
- Subscribe to cable television to watch channels like Fox News, CNN, and The Weather Channel
- Residents prefer vitamins to increase their mileage and a regular exercise regimen

LifeMode 10 Rustic Outposts

- Country life with older families in older homes
- Rustic Outposts depend on manufacturing, retail and healthcare, with pockets of mining and agricultural jobs
- Low labor force participation in skilled and service occupations
- Own affordable, older single-family or mobile homes; vehicle ownership, a must
- Residents live within their means, shop at discount stores and maintain their own vehicles (purchased used) and homes
- Outdoor enthusiasts, who grow their own vegetables, love their pets and enjoy hunting and fishing
- Technology is cost prohibitive and complicated. Pay bills in person, use the yellow pages, read newspapers, magazines, and mail-order books

LifeMode 11 Midtown Singles

- Millennials on the move—single, diverse, urban
- Millennials seeking affordable rents in apartment buildings
- Work in service and unskilled positions, usually close to home or public transportation
- Single parents depend on their paycheck to buy supplies for their very young children
- Midtown Singles embrace the Internet, for social networking and downloading content
- From music and movies to soaps and sports, radio and television fill their lives
- Brand savvy shoppers select budget friendly stores

LifeMode 12 Hometown

- Growing up and staying close to home; single householders
- Close knit urban communities of young singles (many with children)
- Owners of old, single-family houses, or renters in small multi-unit buildings
- Religion is the cornerstone of many of these communities
- Visit discount stores and clip coupons, frequently play the lottery at convenience stores
- Canned, packaged and frozen foods help to make ends meet

| |
|---|
| • Purchase used vehicles to get them to and from nearby jobs |
| LifeMode 13 Next Wave<br><br>• Urban denizens, young, diverse, hard-working families<br>• Extremely diverse with a Hispanic majority, the highest among LifeMode groups<br>• A large share are foreign born and speak only their native language<br>• Young, or multigenerational, families with children are typical<br>• Most are renters in older multi-unit structures, built in the 1960s or earlier<br>• Hard-working with long commutes to jobs, often utilizing public transit to commute to work<br>• Spending reflects the youth of these consumers, focus on children (top market for children's apparel) and personal appearance<br>• Also, a top market for movie goers (second only to college students) and fast food<br>• Partial to soccer and basketball |
| LifeMode 14 Scholars and Patriots<br><br>• College and military populations that share many traits due to the transitional nature of this LifeMode Group<br>• Highly mobile, recently moved to attend school or serve in military<br>• The youngest market group, with a majority in the 15 to 24-year-old range<br>• Renters with roommates in nonfamily households<br>• For many, no vehicle is necessary as they live close to campus, military base or jobs<br>• Fast-growing group with most living in apartments<br>• Part-time jobs help to supplement active lifestyles<br>• Millennials are tethered to their phones and electronic devices, typically spending over 5 hours online everyday tweeting, blogging, and consuming media<br>• Purchases aimed at fitness, fashion, technology and the necessities of moving<br>• Highly social, free time is spent enjoying music, being out with friends, seeing movies<br>• Try to eat healthy, but often succumb to fast food |
